# Supplementary material for: Genome-wide association and transcriptome studies identify target genes and risk loci for breast cancer
Source: Nat Commun. 2019 Apr 15;10:1741. doi: 10.1038/s41467-018-08053-5 (PMC6465407; doi:10.1038/s41467-018-08053-5)
Supplement: Supplementary file 25 — Description of Additional Supplementary Files [file 41467_2018_8053_MOESM25_ESM.docx]

Description of Additional Supplementary Files

Supplementary Data 1. Variants independently associated with breast cancer risk in the joint association analysis (P<5x10-8).

Supplementary Data 2. Genome-wide association studies of gene expression levels queried to identify sentinel eQTL.

Supplementary Data 3. Predicted target genes of sentinel risk variants for overall breast cancer.

Supplementary Data 4. Sentinel risk variants for overall breast cancer that are in LD (r2>0.8) with one or more sentinel eQTL.

Supplementary Data 5. Genes identified as likely targets of overall breast cancer risk variants in this study.

Supplementary Data 6. Summary of directional effect of individual sentinel risk variants for overall breast cancer on target gene expression.

Supplementary Data 7. Association between the 88 likely target genes and breast cancer risk, based on the S-PrediXcan analysis, using whole-blood gene expression prediction models from the DGN study (n=922) and the GTEx consortium (n=369).

Supplementary Data 8. Cell types considered when predicting target genes of sentinel risk variants using information from chromatin interactions and enhancer-expression correlations.

Supplementary Data 9. Subset of plausible target genes that were also predicted to be the target of overall breast cancer sentinel risk variants based on (1) the presence of enhancer-promoter chromatin interactions (cHi-C, ChIA-PET or isHi-C); or (2) a significant correlation between enhancer epigenetic marks and gene expression levels (PreSTIGE, Hnisz, IM-PET, FANTOM5).

Supplementary Data 10. Sentinel eQTL included in the EUGENE gene-based analysis of overall breast cancer.

Supplementary Data 11. Association between each sentinel eQTL included in the EUGENE gene-based test and (i) overall breast cancer risk; and (ii) gene expression levels.

Supplementary Data 12. Summary of the directional effect of the disease-protective allele on gene expression, across all disease-associated eQTL, for genes identified at novel loci for overall breast cancer risk.

Supplementary Data 13. Results from EUGENE gene-based analysis for target genes of sentinel risk variants for overall breast cancer.

Supplementary TableData 14. Variants independently associated with ER-negative breast cancer risk in the joint association analysis (P<5x10-8)

Supplementary Data 15. Sentinel risk variants for ER-negative breast cancer in LD (r2>0.8) with one or more sentinel eQTL

Supplementary Data 16. Predicted target genes of sentinel risk variants for ER-negative breast cancer.

Supplementary Data 17. Genes identified as likely targets of ER-negative breast cancer risk variants in this study.

Supplementary Data 18. Summary of directional effect of individual sentinel risk variants for ER-negative breast cancer on target gene expression.

Supplementary Data 19. Subset of plausible target genes that were also predicted to be the target of sentinel risk variants for ER-negative breast cancer based on the presence of

enhancer-promoter chromatin interactions (cHi-C, ChIA-PET or isHi-C) and a significant correlation between enhancer epigenetic marks and gene expression levels (PreSTIGE, Hnisz, IM-PET, FANTOM5).

Supplementary Data 20. Sentinel eQTL included in the gene-based test for genes identified in the EUGENE analysis of ER-negative breast cancer

Supplementary Data 21. Association between each sentinel eQTL included in the EUGENE gene-based test and (i) ER-negative breast cancer risk; and (ii) gene expression levels.

Supplementary Table 22. Summary of the directional effect of the disease-protective allele on gene expression, across all disease-associated eQTL, for genes identified at novel loci for ER-negative breast cancer risk.

Supplementary Data 23. Results from EUGENE gene-based analysis for target genes of sentinel risk variants for ER-negative breast cancer.
