# Supplementary material for: Genome-wide association and transcriptome studies identify target genes and risk loci for breast cancer
Source: Nat Commun. 2019 Apr 15;10:1741. doi: 10.1038/s41467-018-08053-5 (PMC6465407; doi:10.1038/s41467-018-08053-5)
Supplement: Supplementary file 26 — Supplementary Information [file 41467_2018_8053_MOESM26_ESM.pdf]

Genome-wide association and transcriptome studies identify target genes and risk loci for breast cancer

Ferreira et al.

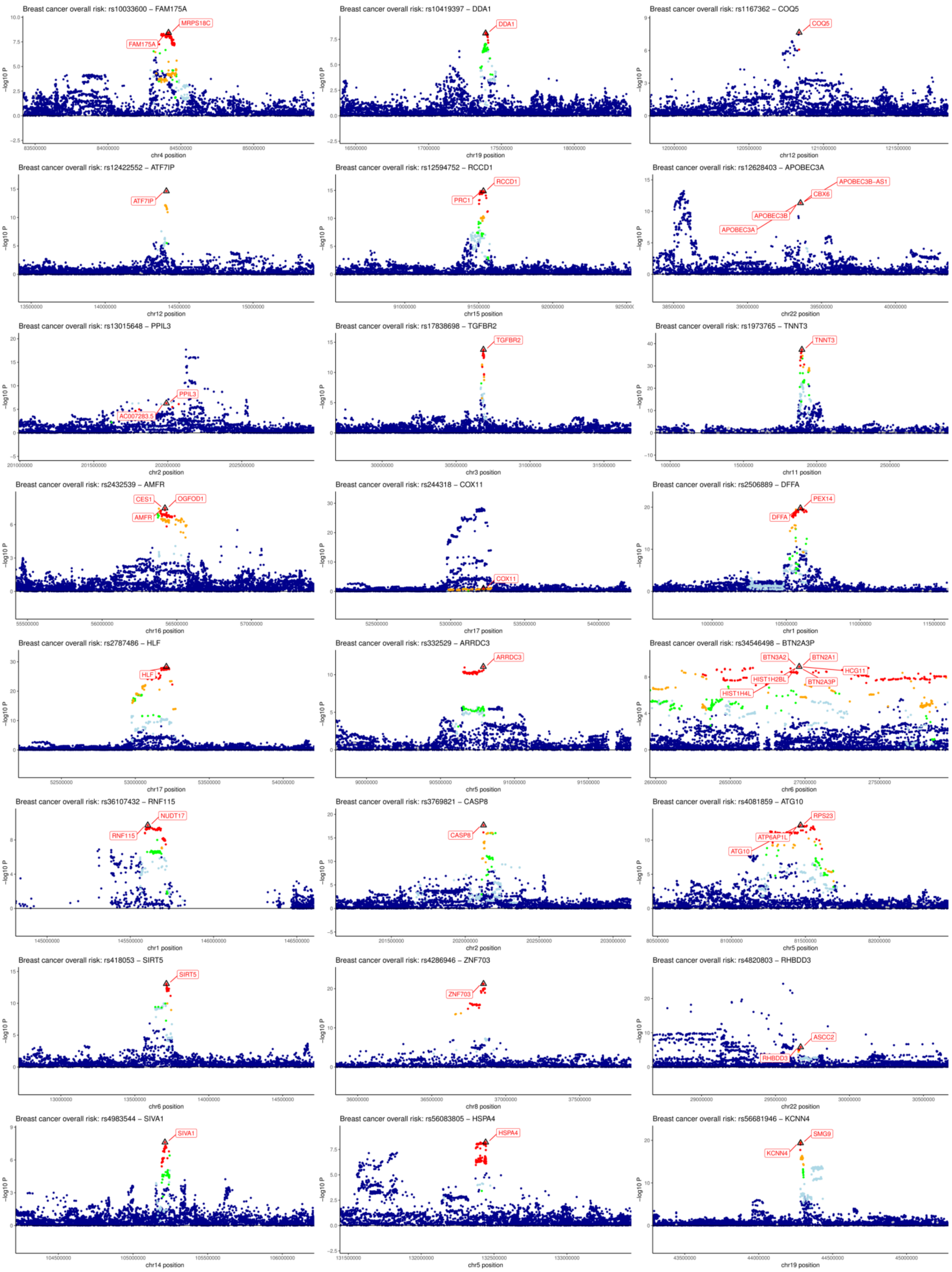
Breast cancer overall risk: rs10033600 – FAM175A
MRPS18C
FAM175A
chr4 position
Breast cancer overall risk: rs10419397 – DDA1
DDA1
chr19 position
Breast cancer overall risk: rs1167362 – COQ5
COQ5
chr12 position
Breast cancer overall risk: rs12422552 – ATF7IP
ATF7IP
chr12 position
Breast cancer overall risk: rs12594752 – RCCD1
RCCD1
PRC1
chr15 position
Breast cancer overall risk: rs12628403 – APOBEC3A
APOBEC3B-AS1
CBX6
APOBEC3B
APOBEC3A
chr22 position
Breast cancer overall risk: rs13015648 – PPIL3
PPIL3
AC007283.5
chr2 position
Breast cancer overall risk: rs17838698 – TGFBR2
TGFBR2
chr3 position
Breast cancer overall risk: rs1973765 – TNNT3
TNNT3
chr11 position
Breast cancer overall risk: rs2432539 – AMFR
CES1
OGFOD1
AMFR
chr16 position
Breast cancer overall risk: rs244318 – COX11
COX11
chr17 position
Breast cancer overall risk: rs2506889 – DFFA
PEX14
DFFA
chr1 position
Breast cancer overall risk: rs2787486 – HLF
HLF
chr17 position
Breast cancer overall risk: rs332529 – ARRDC3
ARRDC3
chr5 position
Breast cancer overall risk: rs34546498 – BTN2A3P
BTN3A2
BTN2A1
HCG11
HIST1H2BL
BTN2A3P
HIST1H4L
chr6 position
Breast cancer overall risk: rs36107432 – RNF115
NUDT17
RNF115
chr1 position
Breast cancer overall risk: rs3769821 – CASP8
CASP8
chr2 position
Breast cancer overall risk: rs4081859 – ATG10
RPS23
ATP6AP1L
ATG10
chr5 position
Breast cancer overall risk: rs418053 – SIRT5
SIRT5
chr6 position
Breast cancer overall risk: rs4286946 – ZNF703
ZNF703
chr8 position
Breast cancer overall risk: rs4820803 – RHBDD3
ASCC2
RHBDD3
chr22 position
Breast cancer overall risk: rs4983544 – SIVA1
SIVA1
chr14 position
Breast cancer overall risk: rs56083805 – HSPA4
HSPA4
chr5 position
Breast cancer overall risk: rs56681946 – KCNN4
SMG9
KCNN4
chr19 position
–log10 P

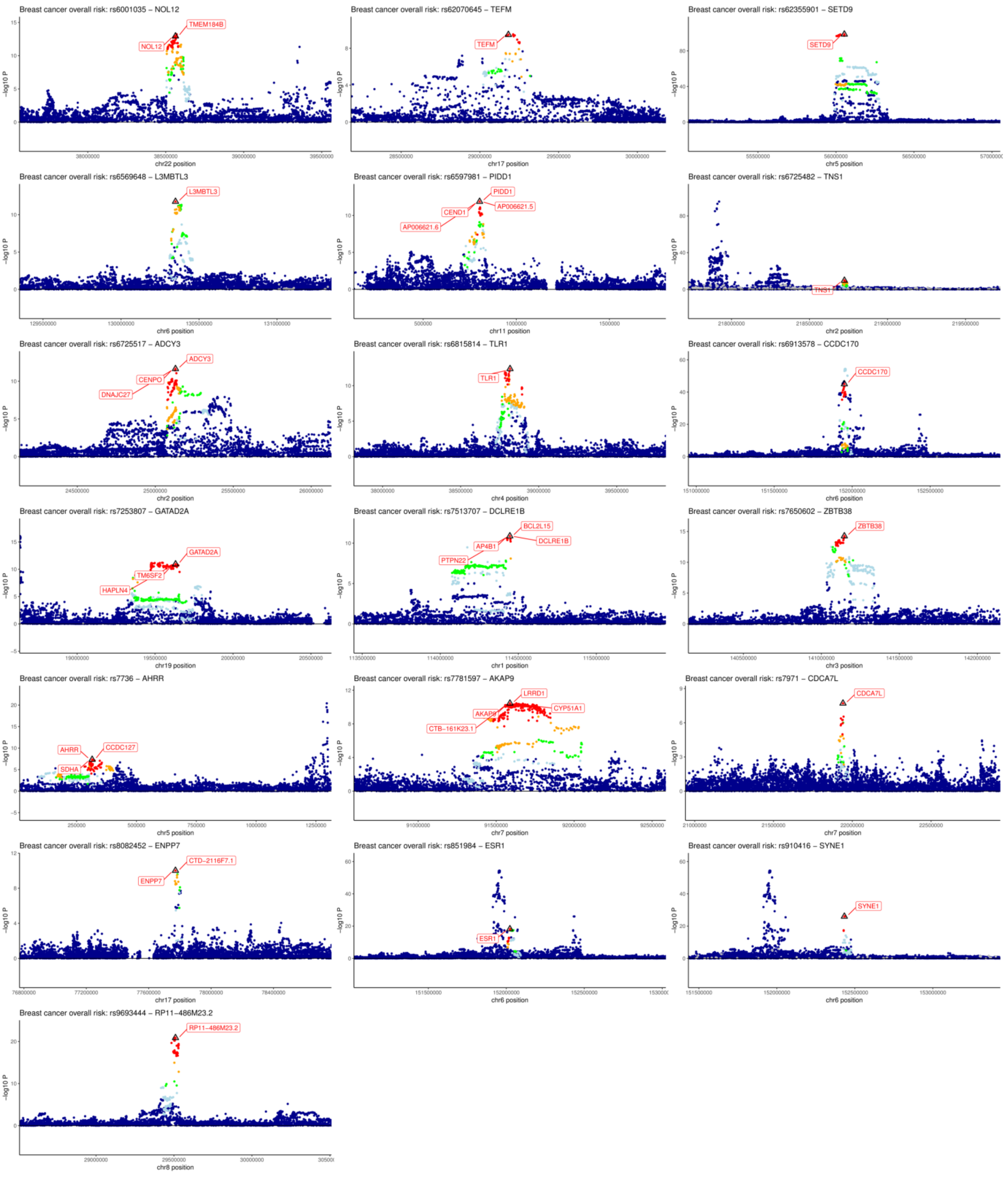

Supplementary Figure 1. Previously unreported target gene predictions at known risk loci for overall breast cancer.

Variants are represented by points coloured according to the LD with the sentinel risk variant (red: >=0.8, orange: 0.6-0.8, green: 0.4-0.6, light blue: 0.2-0.4, and dark blue: <0.2). Sentinel risk variants (triangles) were identified based on joint association analysis [1]. Figure shows on the y-axis the evidence for breast cancer association (-log10 of the P-value in the original published GWAS results [2]), and on the x-axis chromosomal position.

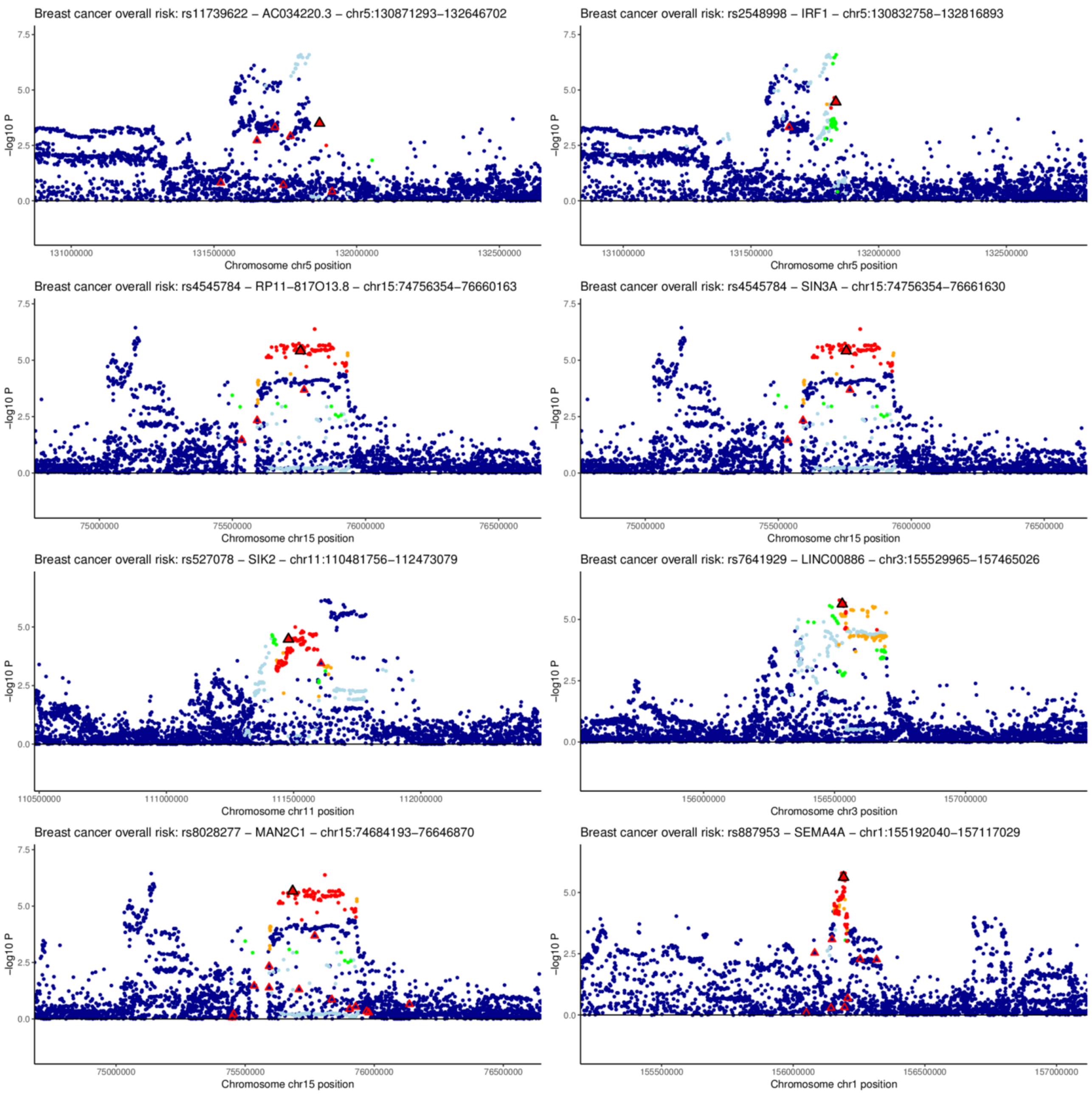

Supplementary Figure 2. Significant gene-based associations at previously unreported risk loci for overall breast cancer.

Variants are represented by points coloured according to the LD with the sentinel risk variant (red: >=0.8, orange: 0.6-0.8, green: 0.4-0.6, light blue: 0.2-0.4, and dark blue: <0.2). Sentinel eQTL included in the EUGENE analysis (triangles) were identified from published eQTL studies of five different tissue types. Figure shows on the y-axis the evidence for breast cancer association (-log10 of the P-value in the published GWAS after adjusting for the association with the sentinel risk variants), and on the x-axis chromosomal position. The sentinel eQTL most associated with breast cancer risk is depicted by a black triangle; other sentinel eQTL are depicted by red triangles.

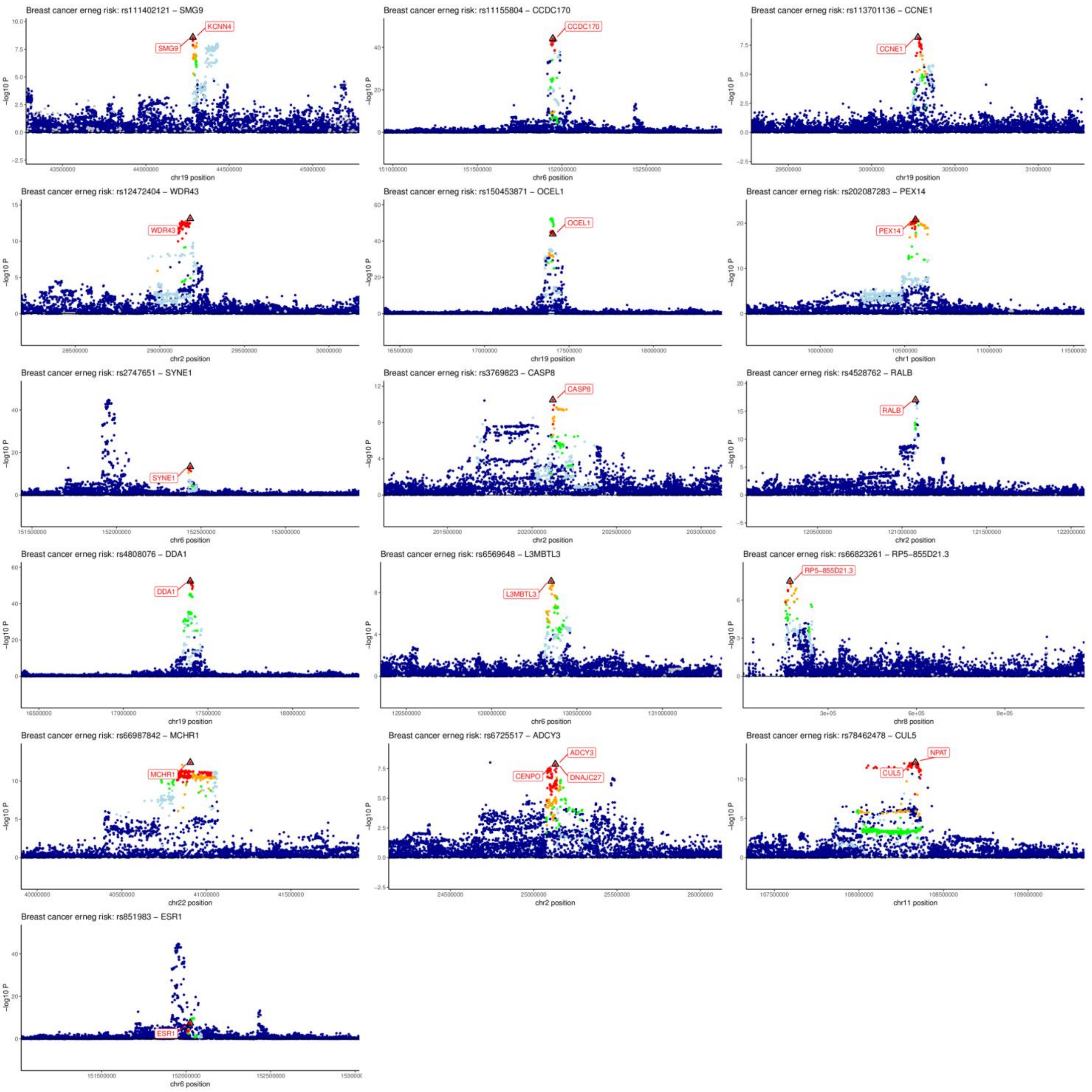

Supplementary Figure 3. Previously unreported target gene predictions at known risk loci for ER-negative breast cancer.

Variants are represented by points coloured according to the LD with the sentinel risk variant (red: >=0.8, orange: 0.6-0.8, green: 0.4-0.6, light blue: 0.2-0.4, and dark blue: <0.2). Sentinel risk variants (triangles) were identified based on joint association analysis [1]. Figure shows on the y-axis the evidence for breast cancer association (-log10 of the P-value in the original published GWAS results [3]), and on the x-axis chromosomal position.

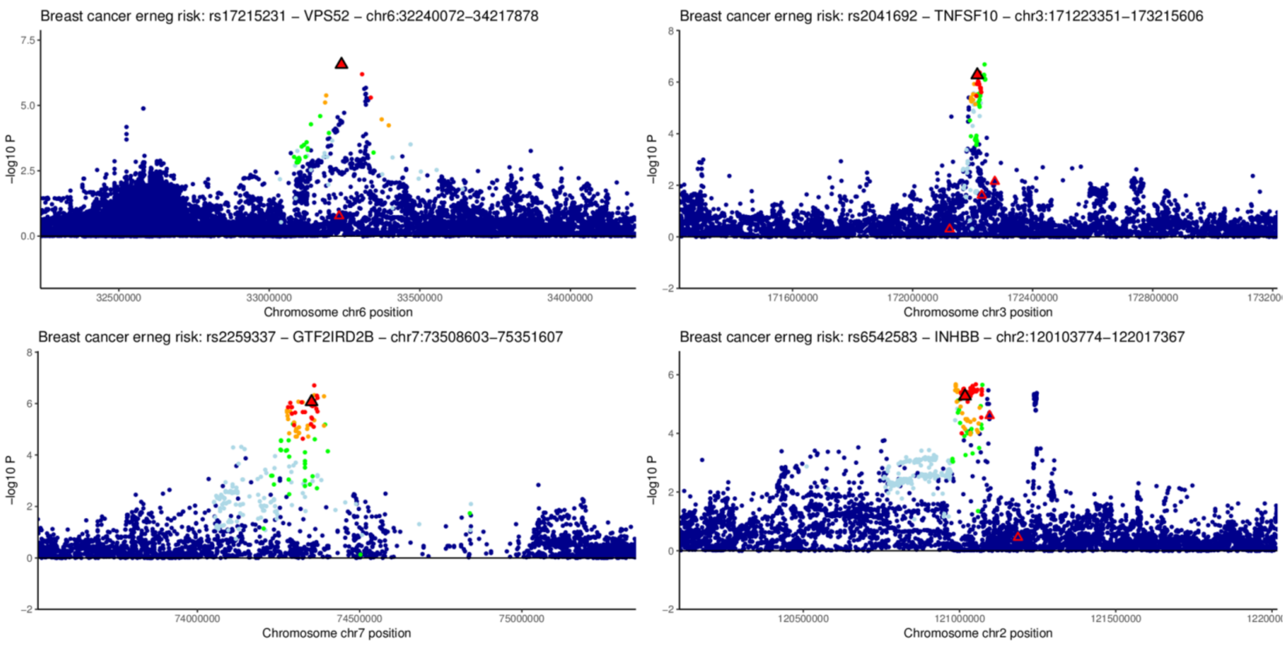

Supplementary Figure 4. Significant gene-based associations at previously unreported risk loci for ER-negative breast cancer.

Variants are represented by points coloured according to the LD with the sentinel risk variant (red: >=0.8, orange: 0.6-0.8, green: 0.4-0.6, light blue: 0.2-0.4, and dark blue: <0.2). Sentinel risk variants (triangles) were identified based on joint association analysis [1]. Figure shows on the y-axis the evidence for ER-negative breast cancer association (-log10 of the P-value in the original published GWAS results [3]), and on the x-axis chromosomal position.

Supplementary References

1. Yang, J. *et al.* Conditional and joint multiple-SNP analysis of GWAS summary statistics identifies additional variants influencing complex traits. *Nat Genet* **44**, 369-75, S1-3 (2012).
2. Michailidou, K. *et al.* Association analysis identifies 65 new breast cancer risk loci. *Nature* **551**, 92-94 (2017).
3. Milne, R.L. *et al.* Identification of ten variants associated with risk of estrogen-receptor-negative breast cancer. *Nature Genet* **49**, 1767-1778 (2017).
